# Paper promises: Peruvian frontline health workers’ perspectives on mental health policies during COVID-19

**DOI:** 10.1093/heapol/czad055

**Published:** 2023-11-16

**Authors:** Nikol Mayo-Puchoc, Jenny Bejarano-Carranza, Rubí Paredes-Angeles, Ana Lucía Vilela-Estrada, Jackeline García-Serna, Noelia Cusihuaman-Lope, David Villarreal-Zegarra, Victoria Cavero, Sara Ardila-Gómez

**Affiliations:** Instituto Peruano de Orientación Psicológica, 208 Manuel Corpancho Av, Lima 15046, Peru; Instituto Peruano de Orientación Psicológica, 208 Manuel Corpancho Av, Lima 15046, Peru; CRONICAS Centre of Excellence in Chronic Diseases, Universidad Peruana Cayetano Heredia, 445 Armendáriz Av, Lima 15074, Peru; CRONICAS Centre of Excellence in Chronic Diseases, Universidad Peruana Cayetano Heredia, 445 Armendáriz Av, Lima 15074, Peru; Instituto Peruano de Orientación Psicológica, 208 Manuel Corpancho Av, Lima 15046, Peru; CRONICAS Centre of Excellence in Chronic Diseases, Universidad Peruana Cayetano Heredia, 445 Armendáriz Av, Lima 15074, Peru; Instituto Peruano de Orientación Psicológica, 208 Manuel Corpancho Av, Lima 15046, Peru; CRONICAS Centre of Excellence in Chronic Diseases, Universidad Peruana Cayetano Heredia, 445 Armendáriz Av, Lima 15074, Peru; Consejo Nacional de Investigaciones Científicas y Técnicas (CONICET), Godoy Cruz 2290, Buenos Aires 1425, Argentina; Instituto de Investigaciones, Facultad de Psicología, Universidad de Buenos Aires, Lavalle 2353, Buenos Aires 1052, Argentina

**Keywords:** Health workers, mental health, health policy, qualitative research, Peru

## Abstract

Governments globally deployed various non-pharmacological public health measures to respond to the COVID-19 pandemic (i.e. lockdowns and suspension of transportation, amongst others); some of these measures had an influence on society’s mental health. Specific mental health policies were therefore implemented to mitigate the potential mental health impact of the pandemic. We aimed to explore the implementation of mental health regulations adopted by the Peruvian health system by focusing on the care services at Community Mental Health Centres (CMHCs), based on the experiences of health workers. We conducted a phenomenological qualitative study to understand the implementation of mental health policies launched in Peru during the COVID-19 pandemic. Data were obtained from a document review of 15 national policy measures implemented during the pandemic (March 2020 to September 2021), and 20 interviews with health workers from CMHCs (September 2021 to February 2022). The analysis was conducted using thematic content analysis. Most implemented policies adapted CMHC care services to a virtual modality during the COVID-19 pandemic; however, various challenges and barriers were evidenced in the process, which prevented effective adaptation of services. Workers perceived that ineffective telemedicine use was attributed to a gap in access to technology at the CMHCs and also by users, ranging from limited access to technological devices to a lack of technological skills. Further, although mental health promotion and prevention policies targeting the community were proposed, CMHC staff reported temporary interruption of these services during the first wave. The disparity between what is stated in the regulations and the experiences of health workers is evident. Policies that focus on mental health need to provide practical and flexible methods taking into consideration both the needs of CMHCs and socio-cultural characteristics that may affect their implementation.

Key messages

- In Peru, the implementation of mental health policies in response to the COVID-19 pandemic was greatly affected by the economic and social context prior to the pandemic.
- CMHC services targeted at the community were temporarily interrupted due to face-to-face restrictions during the first wave of the pandemic.
- The barriers that workers experienced in the implementation of policies illustrate the importance of ongoing policy analysis that considers the needs of the population and the contextual factors that may affect policy implementation.

## Introduction

The pandemic caused by the Severe Acute Respiratory Syndrome Coronavirus 2 (SARS CoV-2) has impacted millions of people and disrupted government systems worldwide. By September 2022, there were approximately 665 deaths and 71 198 cases per 100 000 people, with the number of cases and deaths likely to increase until 2024 (Pettersson *et al.*, 2022). This event had a major impact on people’s mental health and the effects are expected to be felt in the general population in the medium- to long term (Bourmistrova *et al.*, 2022). These effects are related to high levels of anxiety (Hawes *et al.*, 2022), stress (Li *et al.*, 2021; Nelson *et al.*, 2021) and depression (Pérez-Cano *et al.*, 2020; Liao *et al.*, 2021).

Specific policies to improve mental health during the context of the COVID-19 pandemic have been diverse and aimed at benefiting users and health workers (Kola *et al.*, 2021; Zhang and Kim, 2021; Shah *et al.*, 2022; Villarreal-Zegarra *et al.*, 2022). However, evidence on the effect of these regulations is limited, especially in low- and middle-income countries (LMICs) (Villarreal-Zegarra *et al.*, 2022). In particular, Peru was one of the countries able to respond early to the pandemic by (1) declaring a state of sanitary emergency just nine days after the first case of COVID-19 was detected; (2) enacting a set of policies to address the rapid spread of the pandemic; and (3) developing specific mental health policies targeting the general population, health workers and at-risk groups (Gonzales-Castillo *et al.*, 2020; Ministerio de Salud, 2020a–d).

A key component of the specific mental health policies in Peru were those oriented towards the Community Mental Health Centres (CMHCs), which are a key component of the mental health service network in the country (Toyama *et al.*, 2017). However, whether those procedures were implemented as planned, their level of compliance and the barriers to their adoption are unknown. In addition, as well as other health service delivery centres, the care in CMHCs varied according to restrictive measures and demand for attention during the COVID-19 pandemic. During the first month of the pandemic (March 2020), the number of users served at 58 CMHCs suffered a decrease of 22.78% (from 27 085 to 20 915) compared to the previous month. Later this trend gradually increased, reaching high user service rates at the end of the first wave (October 2020 with 37 758 users). On average, the time series analysis reported a steady increase of 703.5 users served in all CMHCs compared to the previous month during the first and second waves of the pandemic (March 2020 to October 2021) (Villarreal-Zegarra *et al.*, 2023). This is because, during the first months of the COVID-19 pandemic, the Peruvian government closed several mental health services completely as a restrictive measure, abruptly affecting the number of visits to CMHCs. By contrast, there was a subsequent increase in attendance at CMHCs, which was due to several factors, such as the mental health policies established from the second month of the pandemic, the use of telemedicine, the gradual return to face-to-face care, and the number of mental health cases, amongst others (Villarreal-Zegarra *et al.*, 2023).

In this context, it is known that developing and implementing health policies require the management of key stakeholders, organizations and institutions (Campos and Reich, 2019), as well as sustainable mechanisms that enable government agencies to work together and develop solutions (Omaswa and Ivey Boufford, 2014). However, the impact of the pandemic highlighted the vulnerability and lack of preparedness of health systems in both LMICs and high-income countries, as well as the lack of resources to respond to an emergency of such magnitude (Anjum *et al.*, 2020; Shuchman, 2020; Panneer *et al.*, 2022). Especially for fragile health systems, the COVID-19 pandemic caused great damage and setbacks in the health-care area (Angrup *et al.*, 2020; Kaye *et al.*, 2021). In light of this, the implementation of mental health strategies has become an essential step to understanding the capacity of response from governments to COVID-19 (Goldman *et al.*, 2020; Kola *et al.*, 2021; Sacco and De Domenico, 2021) due to the direct consequences of the pandemic on mental health (Huarcaya-Victoria, 2020; Atencio-Janela *et al.*, 2021). In this study, we aimed to explore the implementation of mental health policies introduced by the Peruvian health system that focused on the care services of Community Mental Health Centres (CMHCs), based on the experiences of health workers, because they were present during the implementation process and are able to provide better recommendations.

## Materials and methods

### Study setting

The health system of Peru is fragmented (Ligia *et al.*, 2012), and is composed of public and private health services (Alcalde-Rabanal *et al.*, 2011). Since the approval of the mental health reform in 2012, Peruvian policies have allowed the implementation of CMHCs for mental health care. By 2022, there were 229 CMHCs in operation, attending to more than 360 000 cases per year throughout the regions of Peru (Díaz, 2022).

**Figure 1.**
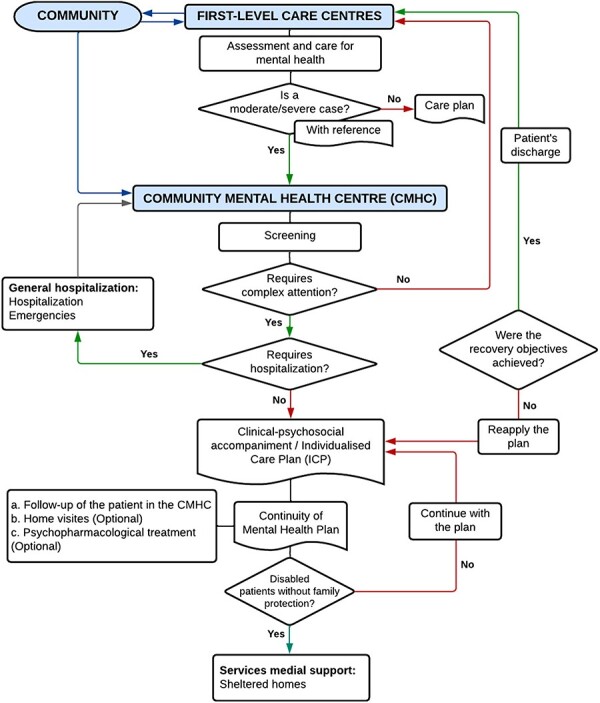
Workflow diagram of the process of care, referral and counter-referral in the CMHC. Source: Adapted from Technical Health Standard. Community Mental Health Centers (RM N° 574–2017/MINSA). https://www.gob.pe/es/i/279706

The CMHCs seek to improve access to health services for people with mental health disorders, psychosocial problems, and their families and local communities. The main functions of the CMHCs are: (1) technical assistance, monitoring and training for workers at first-level health-care centres; (2) specialized outpatient care to users with mental health problems, referred from first-level care centres or general hospitals; and (3) social activities to improve the mental health of users, families and community, such as mental health promotion programmes against the stigma of mental health disorders in the community, amongst others. The CMHC consists of an interdisciplinary team of health and social science professionals (i.e. psychiatrist, psychologist, nurse, medical technician, social worker and nursing technician) (Ministerio de Salud, 2017). Figure 1 shows the care flow of the CMHCs.

### Study design

This qualitative study used a phenomenology approach that sought to understand complex phenomena through the perspectives of participants (Sandelowski and Barroso, 2003). Our study was conducted following the Consolidated Criteria for Reporting Qualitative Research (COREQ) (Booth *et al.*, 2014). The design allowed us to understand how CMHC policies were implemented during COVID-19, by exploring document analysis and individual experiences of health workers during this period.

### Data collection methods

#### Document review

The document review focused on documents that contained government actions especially directed at CMHCs. In total, 15 documents were selected, out of which 7 were issued by the Ministry of Health (MoH) and 8 by Regional Health Directorates (see details in Supplementary Material 1). The documents were gathered from the database of a scoping review conducted by Cavero *et al*. (https://osf.io/tf9h6/), which retrospectively analysed policies related to the mental health response of the Peruvian health systems during the COVID-19 pandemic.

#### Health workers’ interviews

We conducted a secondary analysis from a qualitative study that is part of a larger ongoing project of CRONICAS Centre of Excellence in Chronic Diseases located in Peru, which aims to analyse the care services provided at CMHCs during the COVID-19 pandemic.

The study took place at four CMHCs: three located in Lima, capital of Peru, and one located in Callao, a province adjacent to Lima. These represent approximately 29.9% (10.4 of 33.4 million) of the Peruvian population and 21% (53 of 248 centres) of CMHCs in Peru (Instituto Nacional de Estadística e Informática, 2022; Gobierno del Perú, 2023). The sample was determined using a snowball technique, by asking workers who agreed to interviews to recommend other potential workers for interviews. In this paper, we report the findings of the 20 semi-structured interviews conducted with CMHC workers between September 2021 and February 2022. Participants were health-care and administrative workers from the participating CMHCs. The inclusion criteria were: (1) age 18 years or more, and (2) employed at the participating CMHCs between March 2019 and March 2021. People who started working at the CMHC after the quarantine in Peru had started (16 March 2020) were excluded. The main characteristics of the sample are included in Table 1.

**Table 1.** Demographic characteristics of the sample

Respondents	*N* (%) or mean ±SD
*N*	20
Age (in years)	42.29 ± 9.98
Sex	
Men	4 (20%)
Women	16 (80%)
Level of seniority	
Head of centre	4 (20%)
Health-care worker	8 (40%)
Administrative workers	8 (40%)
Community Mental Health Centres:	
Centre 1	5 (25%)
Centre 2	5 (25%)
Centre 3	5 (25%)
Centre 4	5 (25%)

Two interview guides were developed by the research team: one for health-care workers (e.g. psychologists, nurses), and another for administrative workers (e.g. admission, pharmacy), with specific questions for heads of CMHCs. These guides included four main sections: (1) characteristics of participants, (2) participants’ perspectives about changes in the care provided by CMHCs, (3) participants’ views about barriers and enablers in the care offered at CMHCs, and (4) participants’ experiences of how the government measures taken to address the COVID-19 pandemic affected their mental health. Interviews were audio-recorded and transcribed verbatim, with each participant assigned a unique ID code. The interviews were thoroughly analysed by a group of authors and after discussion among the team, a coding framework was obtained based on a set of themes that summarized the main issues raised by the health workers.

### Data analysis

First, a document analysis was carried out to describe and categorize the content of the policies, while a classification of codes was done in order to establish the key topics in the study. Table 2 shows an overview of the themes and codes of the analysis stages. As the next step, a thematic framework analysis approach was used to analyse both sources. An inductive coding strategy was conducted to allow inclusion of emerging themes and deductive coding was used to examine the research questions and topic guides (Ritchie *et al.*, 2003). Data were analysed by two researchers and supported by another two researchers. Discussions were held to establish themes and sub-themes, revisiting the transcripts or matrixes until consensus was reached across and within the research team (Creswell, 2013). Supporting quotes were extracted from the interviews to contextualize the identified themes.

**Table 2.** Overview of themes and prominent codes

Stage 1:	Stage 2:
*Content analysis*	*Thematic coding*
Themes	Key categories:
Care services at CMHCs Technical assistance, supervision and training for health centres Specialized outpatient care for people with mental health problems Mental health at the community level	Services provided before the pandemic Use of technology in care services at CMHCs before the pandemic Changes in care based on measures generated in the pandemic context Barriers and difficulties to care during the pandemic Services provided at CMHCs Population groups with the highest demand for care during the pandemic context COVID-19 protocols in place at CMHCs Strategies and facilitators to address difficulties CMHC organization to provide care during the pandemic

CMHCs, Community Mental Health Centres.

### Ethics

The protocol was approved by the institutional ethics committee of the authors’ institute in Lima, Peru (ID 203 096). All participants were given information about the study and signed a consent form prior to participation.

## Results

**Figure 2.**
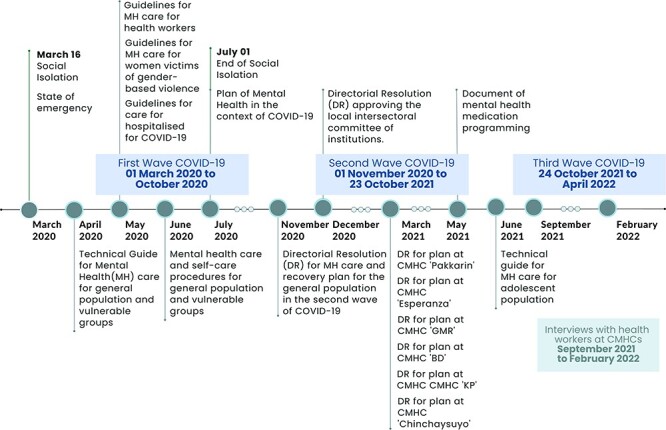
Timeline of COVID-19 response measures implemented during 2020–21 relative to this study’s data collection

Figure 2 shows a timeline designed in an attempt to describe and highlight the main events that occurred in Peru between March 2020 and February 2022, reflecting the mental health policies enacted in relation to the study and interviews with CMHC workers. In this study, two themes were identified based on the CMHCs’ functions: (1) specialized outpatient care for people with mental health problems (six sub-themes); and (2) mental health at the community level.

### Specialized outpatient care for people with mental health problems

#### Prevention and self-care measures for workers

By May 2020, the MoH had published a response plan on the self-care of health workers in order to improve their mental health, outlining several measures to support workers’ self-care and important strategies to prevent mental health issues among the workers at CMHCs (Ministerio de Salud, 2020a). Following the MoH recommendations, interviewees highlighted that CMHC workers implemented leisure activities, active breaks and group outings during the pandemic.

*We worked on behavioural activation through spontaneous activities and active breaks. So, we did some activities, we played bingo, we had some relaxing moments to ease a bit the saturation of responsibilities.* (Head of CMHC 3)

During the COVID-19 pandemic, employees noted that once cases of COVID-19 decreased, there was increased demand from users who turned up at the CMHCs. In this context, workers from two centres noted that employment rights were guaranteed as much as possible (i.e. bereavement leave, vacations and maintaining the workers’ employment contracts). However, there were also worker resignations due to the heavy workload and lack of organization in the provision of their employment rights (i.e. lack of rest breaks, failure to provide work bonuses, and non-compliance with schedules at CMHCs), especially during the first wave of the COVID-19 pandemic (March to October 2020).

*At the level of CMHC higher direction, the issue of the workers’ contract should be maintained, I think that offers emotional stability to be able to work… Lately [September 2021] I noticed a lack of organization in the contracts of workers.* (Head of CMHC 1)

*The first wave has been quite harsh, for example, the parents of three colleagues died from COVID-19 [...] They had their leave, but they were very bad, very sad, working because they had to, and sometimes, they cried.* (Administrative worker #1 at CMHC 3)

Furthermore, a policy on the development of a series of strategies to promote resilience and reduce the risk of developing mental health problems was issued. It proposed activities such as awareness-raising group sessions, training and information brochures, amongst others (Ministerio de Salud, 2020a). During the first wave, respondents emphasized facing increasing challenges to their mental health as a result of the high demand for care at CMHCs. Many interviewees mentioned that CMHC staff conducted strategies to manage by their own means the emotional burden they faced rather than from MoH resources. They listed group self-care meetings, relaxation sessions and open conversations to cope with this burden. Once the mental health self-care guide for workers was published, by order of the MoH, mental health training for health workers was added.

Interviewer: *And the initiative of self-care spaces comes from the MoH or the CMHC’s higher direction?*

Interviewee: *No, it was our initiative precisely from the fact that many workers broke down [...] they collapsed at the point of crying because they could no longer handle the pain due to the cases of violence in girls, adolescents.* (Head of CMHC 2)

#### Screening and assessment of mental disorders and psychosocial problems

Policies focused on maintaining a process of active identification of mental health problems. One policy recommended a process of stages from an initial interview to the application of screening instruments (i.e. Self-Reporting Questionnaire or SRQ) (Ministerio de Salud, 2020b–d). Workers argued that their usual process of screening for mental health problems had to be adapted to a hybrid modality during the first wave and part of the second wave. Videoconferences and phone calls were implemented, the latter being the most frequently used method, and face-to-face screening was maintained only in specific cases. Also, due to the increasing demand by users at the beginning of the second wave (October 2020), the MoH decided that CMHCs had to provide care to all users and, based on their screening, determine whether to treat them or refer them to other centres of lesser complexity (i.e. primary care centres). Thus, cases requiring a more specialized approach were referred to psychological and/or psychiatric intervention. In the middle of the second wave (September 2021), face-to-face screening returned and remote screening was maintained only for vulnerable patients.

*In the first trimester of the pandemic, the care service was provided in a hybrid modality between virtual and some face-to-face activities. Afterwards, we changed to face-to-face care except for specific cases that are still done virtually, such as a user with physical comorbidity that does not allow them to leave home.* (Health-care worker #1 at CMHC 2)

The MoH also established three policies on the screening process for the mental health problems of specific populations (Ministerio de Salud, 2020a,b,d,e). First, interviewees noted that during the pandemic, there was a strengthening of communication with Women’s Emergency Centres (WECs) in the referral of cases of violence, through a remote modality. Respondents added that if they detected a case of violence at the CMHC without a prior legal complaint, they acted according to the Joint Action Protocol with the WECs, referring the user to the WEC for legal guidance. Second, interviewees noted that intervention with children was carried out remotely but was not prioritized due to low user demand. Third, some workers commented that screening for health workers from other health facilities was conducted by the Psychosocial Accompaniment Team for Health Workers (PATHW) formed by professionals from their CMHCs. The PATHW accompanied professionals with mental health problems identified by the higher direction of CMHCs or by self-referral through the SRQ questionnaire.

*A PATWH was formed to provide emotional support to health workers who were becoming unregulated due to the workload [...] We provided psychosocial accompaniment virtually to specific people... for example, workers who had cared for 10 people and 8 of them died.* (Head of CMHC 3)

#### Clinical-psychosocial accompaniment

In the response plan, the MoH recommended CMHCs coordinate with key persons to help in the psychosocial accompaniment of people with severe mental disorders and/or disability (Ministerio de Salud, 2020c). Interviewees noted that when users were unable to attend the CMHC to receive their medication, other options were coordinated, such as enabling a family member or a neighbour to collect it or hiring a delivery service to transport the medication to their homes.

*If users had comorbidities, we would make an agreement with their relatives so that they could go and collect their medication if needed.* (Health-care worker #1 at CMHC 3)

By April 2020, four policies were introduced on support for users experiencing psychosocial and mental health problems (i.e. acute stress, depression, anxiety and violence against women). A set of recommendations was proposed to develop an intervention plan and how health workers should act in complex cases (i.e. rape) (Ministerio de Salud, 2020c,d). At the beginning of the pandemic (March 2020), face-to-face care at CMHCs was limited to psychiatric emergencies or medication outreach due to social restriction measures. Further, workers reported that several users discontinued their treatments possibly due to fear or not knowing whether the CMHCs continued to function. Nevertheless, they tried to hold consultations remotely, both synchronously (e.g. Zoom, Google Meet) and asynchronously by different means, such as Facebook, WhatsApp, text messaging, and others. However, many respondents claimed limitations in the use of technology. They reported having to use their own phones, internet connectivity was not available or deficient, and computers were not available in all CMHCs. They also added that many users did not have phones or their phones did not support videoconferences, they had no internet connectivity, or they did not know how to use their mobile phones.

*We suffer a lot... it was a recurring request to the Direction to fix the internet, we don’t have telephone lines, not all the offices have computers... it’s quite a serious problem [...] Basically due to the infrastructure, we don’t provide the service correctly.* (Head of CMHC 4)

During the first wave, group activities were also discontinued in most of the centres interviewed, prioritizing attention only to users with severe mental disorders, rehabilitation and victims of violence. Some workers mentioned that the reasons group activities failed to return in their entirety were that: certain activities could not be carried out virtually, some users had no mobile phone equipped to support videoconferences, and the low demand of users due to fear of catching COVID-19.

*The psychosocial club was discontinued because most of the users were vulnerable and not all of them had a mobile phone that could support a transmission. It was also because the activities of the psychosocial club are leisure or therapeutic exercises, such as going out for visits, going to the cinema, going to the park, and doing handicrafts. I mean, peer-to-peer interaction was predominant.* (Head of CMHC 2)

Some policies directed at people infected with COVID-19 were also proposed. They identified different profiles: CMHC users who were infected with COVID-19, COVID-19 survivors with mental or physical sequelae, and relatives of deceased or hospitalized COVID-19 users. CMHCs were to ensure the mental health care of users by telemedicine, seeking to promote users’ well-being through a specialized intervention plan (Ministerio de Salud, 2020b,c,e,f). All respondents reported that the care service provided by the CMHC was remote (e.g. phone calls) during the pandemic. They further explained that psychosocial accompaniment was structured based on the needs of the COVID-19 user and was closely monitored because of their health condition. In addition to this, one worker commented that although during 2020 care was made more flexible for all users who required it, from the second wave onwards, care was gradually restricted to mild cases of COVID-19 in order to prioritize moderate and severe cases of COVID-19.

The COVID-19 users who attended the CMHC presented mental health problems related to severe psychological distress, dysregulation due to adjustment or bereavement issues, and habit-forming behaviours. For the psychosocial accompaniment of family members of deceased COVID-19 users, the focus was on bereavement processes and emotional support for the whole family.

*We have supported people who have experienced COVID-19 and we have also supported the grieving process of these people, everything that represents the ritual of the non-physical farewell because many people were not able to say goodbye to their relatives and this generates an ambiguous loss.* (Head of CMHC 3)

For users with a mild COVID-19 condition, the focus was on providing guidelines on protective measures at home or leisure activities to manage the days of isolation they experienced.

*So, the follow-up was done according to the protection measures that the users had to have at home, to look for recreational alternatives, among other issues. The monitoring gave guidelines and/or measures so that the user would feel a little quieter, and could carry out the process as well as possible. Those users with anxiety, however, were automatically referred to psychology.* (Health-care worker #2 at CMHC 3)

Additionally, workers stated that they continued to provide psychosocial support to health workers. In this line, the MoH established two regulations recommending that care of workers be more personalized and focused on their individual needs (Ministerio de Salud, 2020a,e). Thus, interviewees reported that, during the pandemic, they provided care to health professionals from other facilities who requested care directly or were referred to the CMHCs by the PATHW.

*There was an indication from the MoH to work on bereavement in relation to the centres. We have, as part of the technical norm, psychosocial accompaniment for the centres for identification and referral in the case of the user no longer being able to be managed by them. The centre is more specialized in cases of depression, anxiety and schizophrenia, among others.* (Health-care worker #2 at CMHC 1)

#### Continuity of mental health services in community care networks

The MoH decreed three regulations related to the follow-up provided by the CMHCs as part of their Continuity of Services Programme (CSP) (Ministerio de Salud, 2020b–d). The discontinuation of home visits varied across the centres. In some CMHCs it was suspended and/or adapted to a remote modality at the beginning of the pandemic, but face-to-face activities were reintroduced due to absenteeism of critical users. A worker argued that possible explanations may be that some users had no access to the internet, or did not know how to use other technological devices.

*Screening began to be carried out virtually and home visits were virtual, because we made them through videoconference with the user, with Zoom.* (Health-care worker #1 at CMHC 1)

#### Referral and counter-referral of moderate cases

Seven policies on the referral process provided by CMHCs were identified. By technical regulation, CMHCs only attended to users who were referred from other health centres (i.e. hospitals) for specialized care (Ministerio de Salud, 2020a,c–f). At the beginning of the pandemic (March 2020), all interviewees stated that referrals were made more ‘flexible’, and they attended to anyone who approached the CMHC. These guidelines outlined the active referral and/or counter-referral process for victims of violence, children, adolescents, health workers and COVID users (i.e. new users, regular users, and family members).

*Referrals continued to be maintained because users came from health centres that were not mental health centres in both modalities [face-to-face and remote].* (Head of CMHC 4)

In the middle of the second wave (September 2021), referral care was resumed in the CMHCs, and there was a decrease in referrals of COVID users. Referrals were made from: (1) the CMHC’s higher direction, who regularly prepared a list of COVID users to be reached (i.e. people who lost a relative due to COVID-19); (2) public institutions (i.e. Ministry of Justice, WECs, Ministry of Public Prosecution, regional governments and police stations); (3) MoH telephone lines (i.e. lines 100 and 113 were special hotlines designed to solve health issues among the general population); and (4) health centres (i.e. hospitals, primary care centres). One worker added that if a user came to the CMHC physically harmed, they were automatically referred to a hospital to receive urgent care for their physical injuries.

*[S]ome users have arrived in a critical situation, so I have seen that the nursing technician put blisters on them [...] I have also seen users who have been taken to a hospital but not because of a mental issue, but because they have had certain physical injuries, apparently quite serious… They were taken to the hospital as an emergency. A nursing technician and a social worker had to take her to the hospital.* (Administrative worker #1 at CMHC 3)

#### Pharmacological treatment

In the response plan, the MoH also established regulations for the pharmacological treatment of users at CMHCs. An identified policy was to obtain users’ informed consent before receiving pharmacological treatment, except in cases of psychiatric emergency (Ministerio de Salud, 2020c,e). According to interviewees, pharmacological treatment was one of the most frequently used measures to maintain continuity of services during the pandemic. The use of medication was based on the needs of the user and monitored by the CMHCs during the pandemic. In addition, in cases where a user attending the CMHC became decompensated, a worker reported that an injection was given immediately to calm them down.

*For example, the virtual care was provided by the psychiatrist, who gave the approval and left the prescription at the pharmacy for the user to collect the medicine.* (Health-care worker #2 at CMHC 4)

Conversely, the collection of medicines or the extension of their prescriptions was maintained throughout the pandemic. To facilitate pharmacological treatment, most workers stated that the ‘electronic prescription’ was used. This consisted of a photo of the physical prescription sent via WhatsApp or Facebook to the user and/or the pharmacy service, where the user or a family member could receive the medicine. Treatment monitoring was done via telemedicine or face-to-face in more severe cases.

Interviewer: *Could you tell me a bit more about how the electronic prescription was collected?*

Interviewee: *Of course, it was done intuitively because it had not been protocolised, those of us who work in health services tried a lot of spontaneity and creativity [...] We took photos of the prescription and sent them to the user by WhatsApp. With that, the user could come and exchange the prescription, thus verifying that they were given the same prescription. The medicines were delivered to the pharmacy.* (Head of CMHC 3)

### Mental health at the community level

In the early months of the pandemic (March to June 2020), MoH regulations indicated that the CMHCs should prioritize mental health prevention and promotion actions in the COVID-19 context (Ministerio de Salud, 2020a–e). In line with this, some regulations established that community activities at CMHCs should be aimed at strategies to promote self-care and prevent mental health issues, such as physical activity, stress management and positive coping skills in the COVID-19 context, amongst others (Ministerio de Salud, 2020b,d,e). Despite these good intentions and plans, interviewees highlighted that community activities stopped during the first wave of COVID-19, returning to face-to-face activities at the beginning of the second wave.

*At the beginning of the pandemic, it wasn’t like that, right? Everything was completely suspended.* (Head of CMHC 4)

Conversely, interviewees acknowledged that using technology made it possible to sustain some policies throughout the pandemic. Social networks facilitated the dissemination of information about mental health and the prevention of COVID-19 to the general population (e.g. mask use and handwashing, and key concepts of depression, anxiety and bereavement) (Ministerio de Salud, 2020c,e).

*If you go to our website you can have access to information about depression, grief, anxiety, gambling addiction in the time of COVID-19... every week… .* (Head of CMHC 1)

Workers noted that digital platforms helped to facilitate inter-institutional coordination among police and prosecutors, centres for young people and adolescents, and educational institutions. This facilitated implementing activities for promoting self-care and prevention of mental health issues among adolescents (Ministerio de Salud, 2020d).

*[M]any institutions have continued to communicate, have continued to work collaboratively, and have been able to sustain the continuity of people’s care. We have had very successful and very pleasant experiences with the Adolescent Guidance Service, the ‘Safe Neighborhood’ programme, the police station, the social actors, and the community agents.* (Head of CMHC 3)

In addition, while CMHCs are responsible for inclusive community activities for people with mental disorders and/or disabilities and there was a policy for their implementation, most of the interviewees highlighted that government regulations prioritized mental health activities focused on COVID-19 (Ministerio de Salud, 2020c).

## Discussion

This study explored the implementation of mental health policies from the perspective of CMHC workers during the COVID-19 pandemic in Peru. Overall, regulations showed an apparent concern and a series of efforts by the MoH to address the mental health needs of the population and CMHC workers. Several policy measures were proposed to reinforce the CMHCs’ care services, mainly in outpatient and community-based services. Participants highlighted enablers and barriers that they experienced during the COVID-19 pandemic. Also, they reported strategies that they implemented to address some policy gaps relating to external factors such as user demand, COVID-19 infections, and challenges to putting the policies into practice.

The majority of strategies that were implemented adapted services provided by the CMHCs to a virtual modality during the pandemic. Responses from the staff placed greater emphasis on use of remote care as a method to deliver psychosocial services to users (e.g. counselling and workshops with the family) and community-level care (e.g. information dissemination, mental health talks and coordination with other institutions). CMHC workers used various modes of telehealth delivery including videoconferences (i.e. Zoom and Meet), websites (i.e. CMHC pages), social networks (i.e. Facebook), instant messaging services (i.e. WhatsApp), and telephone (i.e. phone calls, texts and messages). Further research has identified the benefits of remote care such as accessibility of care, ease of communication between workers and users, and reduction of COVID-19 transmission (Monaghesh and Hajizadeh, 2020; Sklar *et al.*, 2021; Beks *et al.*, 2022). As services moved to a virtual modality, workers also noted several difficulties during the care process. CMHCs were not used to using technological tools; the unavailability of computers and cell phones, poor or unavailable internet connectivity, and the lack of expertise of some CMHC workers in using technological devices were limiting factors. Further, the lack of access and technological knowledge or the limited resources of some service users were also noted as barriers. The perception of increased user demand and in turn increased workload in remote care led to workers’ preference for face-to-face care. The results were consistent with other studies in different health-care settings during the COVID-19 pandemic (Sklar *et al.*, 2021; Rains *et al.*, 2022; Schriger *et al.*, 2022), especially in LMICs (Vigo *et al.*, 2020; Ardila-Gómez *et al.*, 2021; Munthali-Mulemba *et al.*, 2022). One study identified that vulnerable patients (i.e. older users and users with severe mental disorders) are less likely to use telemedicine (Ainslie *et al.*, 2022).

In terms of policies that were not implemented, workers highlighted that services at the community level, such as programmes for the prevention of mental health issues and promotion of mental health care, were suspended during the first wave of the COVID-19 pandemic (March to October 2021). Reasons may be the strategic shift implemented by the MoH in prioritizing essential services. In line with this, the third report of the World Health Organization (WHO) evidenced that in LMICs, face-to-face health services were disrupted, prioritizing provision of health care at hospital facilities to treat COVID-19 patients, leaving aside mental health care. Thereby, public health policies for specific services, such as mental health and community-based services, were the ones most often limited or suspended during the first quarter of the 2021 pandemic (WHO, 2022).

Overall, Peruvian mental health policies prioritized the use of telemedicine for CMHC care. However, our results demonstrate the barriers to effective implementation of routine mental health initiatives. While challenges to implementing new regulations were noted globally, it is important to understand the socio-cultural variations from the point of view of Latin America and Caribbean (LAC) countries. First, mental health reform (i.e. a shift from a biomedical model to a community-based model) had been delayed due to political and economic factors in both HICs and LMICs (Minoletti *et al.*, 2012). Around a third of LACs still do not have a national public mental health policy; moreover, the total budget for mental health policies is lower (2%) compared to HICs (5–10%) (Leiva-Peña *et al.*, 2021; PAHO/WHO, 2022). For example, Peru is one of the countries that has progressively adopted a community-based health system and possesses a national mental health policy (Ministerio de Salud, 2015); however, the budget assigned to mental health is one of the lowest (2%) compared to LACs such as Uruguay (7%), Chile (2.4%) and Venezuela (5%) (Leiva-Peña *et al.*, 2021; Berríos, 2022). This suggests that, although mental health is already a higher priority, the budget allocated remains low even after the pandemic. Legal barriers to implementing telemedicine also remain, as directives have been transposed but do not specify essential aspects such as informed consent, which may affect users’ rights (Presidencia de la República del Perú, 2020; Orellano, 2021).

Second, inequality and poverty continue to be social determinants that pose a major challenge to the design and implementation of public policies in the vast majority of countries in the region (Leiva-Peña *et al.*, 2021). According to the Economic Commission for Latin America and the Caribbean (ECLAC), following the pandemic the rate of poverty increased to 32.1% at the LAC level and 19.3% in Peru, in contrast to 2019 with 30.4% and 15.4%, respectively (Economic Commission for Latin America and the Caribbean, 2023). Consequently, poverty is a stress factor contributing to the development of mental disorders by directly affecting people’s quality of life and increasing the gap in access to the health-care system (Costello *et al.*, 2003; Romero, 2021).

The results of the study acknowledge several strengths and limitations. First, the scoping review and qualitative study were developed by a team of public health experts. Second, the rigorous method of both analyses allowed a broader comprehension of the barriers and challenges between implementing government-issued mental health measures and their actual use in the CMHCs. In contrast, the secondary analysis of the scoping review focused on exploring MoH policies during the first wave of the pandemic (March to September 2020). Thus, the response to new measures that might have influenced the experiences of CMHC workers were not collected. Furthermore, this paper focuses on 20 interviews, a sample that included heads of centres and workers, leaving the perspectives of other groups (i.e. decision-makers and users) unexplored. Additionally, being part of a larger project, the qualitative study did not focus primarily on assessing the policies implemented in the CMHCs but rather was based on workers’ perceptions of CMHC services during the COVID-19 pandemic. Thus the study could not explore in depth policy implementation.

## Conclusion

The Peruvian health system’s response to the COVID-19 pandemic and the policies implemented during the first year have clearly shown that health regulations, especially in mental health, cannot be treated as a low priority. According to Jasanoff (Jasanoff *et al.*, 2021), the COVID-19 pandemic has exacerbated pre-existing conditions and unravelled political values.

Conversely, the use of telemedicine is an effective method of providing access to health care. Although it has been implemented in many countries, implementation in Peru is still a weak process, which has been affected by social and economic factors. Since the mental health reform, mental health policies have evidenced a shift to a community perspective; however, there are still challenges in their operation, financing and adaptation to the socio-cultural context. Hence, while the need to address mental health is already evidenced, and even more because of the COVID-19 pandemic, mental health actions should aim to offer practical methods of implementation and involve actors and institutions that allow for a more comprehensive and effective vision. Future responses to health emergencies should incorporate mental health-focused strategies that allow for the strengthening of primary care in the different mental health services.

## Data Availability

All data related to this article are available upon reasonable request from the corresponding author. Public data, such as policy titles are available within this article and its supplementary material online.

## Abbreviations

LMICs: Low- and Middle-Income Countries

CMHC: Community Mental Health Centre

MoH: Ministry of Health

WECs: Women’s Emergency Centres

PATHW: Psychosocial Accompaniment Team for Health Workers

## Reflexivity statement

All authors are based in the Global South, specifically in the Latin American region. The authors include eight females and one male and span multiple levels of seniority. All authors are involved in research activities focused on Mental Health, Public and Global Health with three authors having several years of experience. Our three senior authors have extensive experience in conducting qualitative research and literature review in the area of Mental Health and Public Health in Latin American and Caribbean countries, especially in Peru and Argentina. Our authorship team also reflects early-career researchers, and all researchers contributed to the analysis and finalization of the manuscript.
